# Clinical Research of Combined Application of DCEUS and Dynamic Contrast-Enhanced MSCT in Preoperative cT Staging of Gastric Cancer

**DOI:** 10.1155/2021/9868585

**Published:** 2021-10-19

**Authors:** Junling Wang, Xia Li, Zhijie Zhang, Chao Jing, Jie Li

**Affiliations:** ^1^Department of Ultrasound, Dezhou People's Hospital, Dezhou, Shandong 253014, China; ^2^Department of Ultrasound, Qilu Hospital of Shandong University, Jinan, Shandong 250012, China

## Abstract

**Purpose:**

To investigate the clinical value of double contrast-enhanced ultrasound (DCEUS) combined with dynamic contrast-enhanced multislice CT (MSCT) in preoperative T staging of gastric cancer (GC).

**Methods:**

206 patients with GC confirmed by preoperative gastroscopy from February 2019 to February 2021 were collected, all patients were examined by DCEUS and dynamic contrast-enhanced MSCT before operation, and the invasion depth (T staging) of GC was evaluated. The diagnosis results of DCEUS, dynamic contrast-enhanced MSCT, and combined diagnosis of DCEUS and MSCT methods (D&M method) were compared with the pathological staging results (gold standard).

**Results:**

The correct diagnosis rate of MSCT was 27.27% in T1 staging, 55.56% in T2 staging, 42.11% in T3 staging, 59.29% in T4 staging, and 55.34% in summation. The correct diagnosis rate of DCEUS was 90.91% in T1 staging, 88.89% in T2 staging, 78.95% in T3 staging, 82.86% in T4 staging, and 83.98% in summation. The correct diagnosis rate of the D&M method was 100.00% in T1 staging, 94.44% in T2 staging, 89.47% in T3 staging, 93.57% in T4 staging, and 93.69% in summation. The D&M method had higher correct diagnosis rate than MSCT or DCEUS alone, the correct diagnosis rate of the D&M method in T1, T2, T3, and T4 staging was significantly higher than that of MSCT (*P* < 0.05). The correct diagnosis rate of the D&M method in T1, T3, and T4 was significantly higher than that of DCEUS (*P* < 0.05). The Youden index of preoperative T1, T2, T3, and T4 staging of GC by the D&M method was 99.49%, 94.44%, 84.13%, and 90.54%, respectively, and the Kappa values of these were 0.954, 0.966, 0.707, and 0.881, respectively.

**Conclusions:**

Dynamic contrast-enhanced MSCT combined with DCEUS in the diagnosis of preoperative cT staging of GC has more validity, reliability, and revenue than the using of MSCT or DCEUS alone, which is an image evaluation method worthy of clinical promotion.

## 1. Introduction

Gastric cancer (GC) is the most common malignant tumor in the world, the incidence rate of malignant tumors is fifth, and the mortality rate is third [1]. At present, although the early diagnosis rate of GC in China is increasing year by year, more than 80% of patients are still in the progressive stage at the first visit. Among all patients with GC who received surgical treatment, the 5-year survival rate of patients with advanced GC was only about 30%, which was significantly lower than that of patients with early GC. Individualized treatment is advocated in patients with early or advanced GC. Accurate evaluation of the clinical staging of GC before formulating the treatment plan is of great significance for the selection of treatment plan and the preliminary evaluation of patients' prognosis. The 8th edition of TNM staging system for GC developed by UICC/AJCC includes pathological staging (pTNM staging), clinical staging (cTNM staging), and pathological staging after neoadjuvant therapy (ypTNM staging) [2]. This system can provide diagnostic basis and theoretical guidance for accurate staging of GC and is of great significance for reasonable selection of treatment options and prognosis evaluation [3]. The common methods for preoperative diagnosis of GC include fiberoptic gastroscopy and histological examination. These methods can make a preliminary diagnosis of GC before operation, but cannot get the tumor staging [4]. There are a lot of clinical research methods for preoperative clinical T staging (cT) of GC, including endoscopic ultrasonography [5], dynamic contrast-enhanced multislice CT (MSCT) [6], magnetic resonance imaging (MRI) [7], and double contrast-enhanced ultrasound (DCEUS) [8]. In this study, DCEUS and dynamic contrast-enhanced MSCT were used for preoperative examination of GC patients and compared with pathological results to explore the application value of the combined application of the two methods in preoperative cT staging of GC.

## 2. Patients and Methods

### 2.1. Clinical Data

The clinical data of 206 patients with GC who were confirmed by gastroscopy before operation, performed abdominal dynamic contrast-enhanced MSCT and DCEUS, underwent radical gastrectomy, and then got the results of pT staging. There were 111 males and 95 females. The age of them ranged from 23 to 81 years old, with an average age of 59.7 ± 11.3 years. This study was approved by the ethics committee of Qilu Hospital of Shandong University. The included patients and their families signed informed consent in advance.

### 2.2. Inclusion Criteria

(1) Preoperative gastroscopy confirmed GC by pathology, excluding distant metastasis of other organs. (2) No other treatment was given before operation. (3) The patients agreed and tolerated radical gastrectomy. (4) MSCT and DCEUS were performed within one week before operation. (5) There was no massive hemorrhage, gastric perforation, or obstruction within 2 weeks before MSCT and DCEUS. (6) The interval between MSCT/DCEUS and the previous biopsy should be more than 3 days. (7) The clinical and pathological data were complete.

### 2.3. Exclusion Criteria

(1) Those who are allergic or contraindicated to anisodamine and/or iodine contrast media and those who are allergic to oral or intravenous contrast media. (2) Whose judgment of cT staging was affected by image artifacts. (3) Poor filling of gastric cavity affects the judgment of cT staging. (4) Those who had hemorrhage, perforation, obstruction, gastric retention, and so on. (5) Patients who received endoscopic resection before operation.

### 2.4. Imaging Equipment and Methods

#### 2.4.1. MSCT

Philips Brilliance 128 row 256 slice spiral CT was used, plain scan and enhanced scan were performed, and the contrast agent was lohexol. Patients should fast for more than 8 hours before examination, drink water 500 ml 30–60 minutes before examination, then intramuscular injection of raceanisodamine hydrochloride injection (produced by Hangzhou Minsheng Pharmaceutical Co., Ltd., 1 ml: 5 mg, H33021707) 10–20 mg, and then drink water 500 ml 15 minutes before examination. The scanning range was from diaphragmatic apex to pubic symphysis. Scanning parameters: 120 kV, 200–250 mAs, pitch 0.938, and collimation 0.625 mm × 128. The contrast agent used in contrast-enhanced scanning was lohexol (iopromide injection, produced by GE Pharmaceutical (Shanghai) Co., Ltd., 100 ml: 30 g (I), H20000595) or ultravist (iopromide injection, produced by Bayer Medical and Health Care Co., Ltd. Guangzhou Branch, 100 ml: 37 g (I), H10970417), with a dose of 1.5 ml/kg body weight and injected through the median cubital vein at a flow rate of 3 ml/s. Low dose test method was used: 16 ml of test dose was injected first, and then the drug was injected in a bolus. The scan was performed in the pulse phase (peak enhancement time was determined by small dose test), portal vein phase (20 s after the arterial phase), and equilibrium phase (60 s after the portal vein phase). The MSCT results and preoperative cT staging evaluation of all patients in this study were performed by two senior doctors in the radiology department of Qilu Hospital of Shandong University.

#### 2.4.2. DCEUS

Acuson Sequoia 512 color ultrasonic diagnostic instrument of Siemens was used. Tianxia brand instant gastrointestinal ultrasound aid (Huzhou East Asia medical supplies Co., Ltd., 50 g/bag, 3230223) was used as an oral contrast agent. Sonovue (sulphur hexafluoride microbubbles for injection, produced by Bracco (Italy) Co., Ltd., 59 mg SF_6_, J20080052) was used as an intravenous contrast agent. Light diet 2-3 days, fasting more than 8 hours, and intramuscular injection of 0.5 mg atropine half an hour before examination, in order to reduce the impact of gastric peristalsis on ultrasound examination. After the oral administration of the contrast agent, the gastric fundus and body were scanned dynamically in real time to observe the size, shape, and scope of lesions. During the examination, the patients were asked to change their position to cooperate with the examination. If necessary, the oral contrast agent could be added to obtain clear images. Intravenous contrast agent was mixed with 5 ml normal saline to form suspension, and 2.4 ml was injected through superficial vein of elbow arm quickly. Then, observation and dynamic recording were started to store the enhancement mode, peak value and duration of the lesion and surrounding normal gastric tissue, gastric wall, and perigastric lymph nodes. The low mechanical index of the linear array probe was 0.07–0.10. The offline analysis software was used to analyze the images and generate the time intensity curve. DCEUS examination and preoperative staging of GC in all patients were performed by two senior doctors in the ultrasound department of Qilu Hospital of Shandong University.

### 2.5. DCEUS Combined with MSCT Image Analysis

The images of the two methods were analyzed by three senior abdominal radiologists (at least 3 years working experience in imaging department) using double-blind method. According to the principle of majority, the cT staging results were obtained.

### 2.6. Criteria for T Staging GC

According to the theory of Kim et al. [5] and referring to the 8th edition of the TNM staging system of GC [3], the criteria of T staging of GC are summarized in Table 1.

### 2.7. Statistical Analysis

SPSS 23.0 software (IBM Corp.) was used for the statistical analysis of the data. The differences were compared with each other *χ*^2^ test, *P* < 0.05 was considered to indicate a statistically significant difference. Kappa consistency test was used to analyze the consistency between preoperative cT staging and postoperative PT staging, 0.75 < *K* ≤ 1 is good consistency, 0.4 < *K* ≤ 0.75 is general consistency, and 0 < *K* ≤ 0.4 is poor consistency. The count data is expressed as rate (%), and the comparison between two groups was made by using the four grid table *χ*^2^ inspection. When *n* ≥ 40 and *t* ≥ 5, Pearson *χ*^2^ test was used for inspection. When *n* ≥ 40 and 1 ≤ *T* < 5, continuous correction was used for inspection.

## 3. Results

### 3.1. Results of MSCT Diagnosis in Preoperative T Staging of GC

In all patients, postoperative pathology was regarded as the “gold standard,” and pT staging of GC included 11 cases of T1, 36 cases of T2, 19 cases of T3, and 140 cases of T4. According to the gold standard, cT staging results of MSCT diagnosis were: 3 cases of T1, 20 cases of T2, 8 cases of T3, and 83 cases of T4. The correct diagnosis rate was 27.27% in T1, 55.56% in T2, 42.11% in T3, 59.29% in T4, and 55.34% in summation. The specific results are shown in Table 2.

### 3.2. Results of DCEUS Diagnosis in Preoperative T Staging of GC

**Figure 1:**
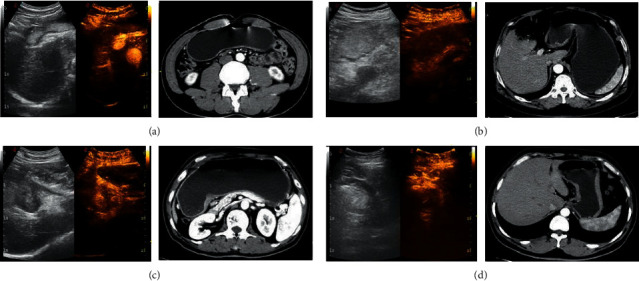
DCEUS image of typical cases. Note: in (a), (b), (c), and (d), the left images were oral gastrointestinal contrast agent ultrasound images, the middle images were double-contrast ultrasound images, and the right images were enhanced MSCT images. (a) An ultrasound image of a 59-year-old male patient; he was finally diagnosed as a moderately differentiated adenocarcinoma of the antrum (pT1 staging). DCEUS showed focal thickening of the gastric wall, with focal positive development of the middle layer in the inner layer of the gastric wall and clear boundary with the outer layer, and it was judged that the tumor infiltrated into the muscularis mucosa. MSCT showed focal gastric wall thickening, and the lesion did not exceed the low-density zone of submucosa (preoperative diagnosis cT1 staging). (b) An ultrasound image of a 60-year-old female patient. She was finally diagnosed as poorly differentiated adenocarcinoma with ulcerative gastric antrum (pT2 staging). DCEUS showed that the whole gastric wall of the lesion was thickened and positively developed, and the outer edge of the lesion was intact and smooth, and it was judged that the tumor infiltrated into the muscle layer; MSCT showed that the gastric wall was thickened, the lesion broke through the slightly strengthened muscle layer of the submucosa, the outer surface of the stomach around the lesion was clear and smooth, and the fat surface around the stomach was clear (preoperative diagnosis of cT2 staging). (c) An ultrasound image of a 64-year-old female patient. She was finally diagnosed as a poorly differentiated adenocarcinoma with an ulcerative type of lesser curvature of stomach (pT3 staging). DCEUS showed that the whole gastric wall of the lesion was obviously thickened and showed positive development, and the outer layer of the tumor was vague and serrated, breaking through the adventitia, but not invading the adjacent structures, and it was judged that the tumor infiltrated into the subserosal layer; MSCT showed gastric wall thickening, irregular fat infiltration around the stomach, and uneven serosal surface (preoperative diagnosis of cT3 staging). (d) An ultrasound image of a 58-year-old male patient. He was finally diagnosed as poorly differentiated adenocarcinoma of the lesser curvature ulcerative type (pT4 staging). DCEUS showed that the whole layer of gastric wall of the lesion was thickened and showed positive development, the tumor invaded the outer serous, and the tumor broke through serosa; MSCT showed that the tumor broke through the perigastric adipose tissue and serosa, accompanied by the expansion and invasion of adjacent organs or structures (preoperative diagnosis of cT4 staging).

According to the gold standard, cT staging results of DCEUS diagnosis were: 10 cases of T1, 32 cases of T2, 15 cases of T3, and 116 cases of T4. The correct diagnosis rate was 90.91% in T1, 88.89% in T2, 78.95% in T3, 82.86% in T4, and 83.98% in summation. The specific results are shown in Table 3, DCEUS and MSCT images of typical cases are shown in Figure 1.

### 3.3. Results of D&M Method Diagnosis in Preoperative T Staging of GC

According to the gold standard, cT staging results of the D&M method diagnosis were: 11 cases of T1, 34 cases of T2, 17 cases of T3, and 131 cases of T4. The correct diagnosis rate was 100.00% in T1, 94.44% in T2, 89.47% in T3, 93.57% in T4, and 93.69% in summation. The specific results are shown in Table 4.

### 3.4. Results of Comparison of Correct Diagnosis Rate of cT Staging

**Figure 2:**
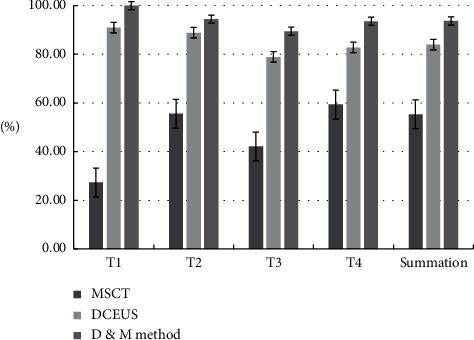
The correct diagnosis rate of T staging results of MSCT, DCEUS, and combined diagnosis. Note: (1) comparison of correct diagnosis rate of MSCT and DCEUS in different T staging: T1 staging, *χ*^2^ = 13.015, *p*=0.000; T2 staging, *χ*^2^ = 6.132, *p*=0.015; T3 staging, *χ*^2^ = 10.451, *p*=0.000; T4 staging, *χ*^2^ = 5.177, *p*=0.019; summation, *χ*^2^ = 7.152, *p*=0.008. (2) Comparison of correct diagnosis rate of MSCT and the D&M method in different T staging: T1 staging, *χ*^2^ = 16.541, *p*=0.000; T2 staging, *χ*^2^ = 9.648, *p*=0.000; T3 staging, *χ*^2^ = 15.224, *p*=0.000; T4 staging, *χ*^2^ = 8.674, *p*=0.004; summation, *χ*^2^ = 11.192, *p*=0.000; (3) Comparison of correct diagnosis rate of DCEUS and D&M method in different T staging: T1 staging, *χ*^2^ = 4.482, *p*=0.034; T2 staging, *χ*^2^ = 2.697, *p*=0.187; T3 staging, *χ*^2^ = 4.847, *p*=0.031; T4 staging, *χ*^2^ = 4.052, *p*=0.044; summation, *χ*^2^ = 2.387, *p*=0.163.

The correct diagnosis rate of MSCT, DCEUS, and D&M method diagnosis of cT staging is shown in Figure 2. The DCEUS method had higher correct diagnosis rate than MSCT method in T1 to T4 staging of GC (*P* < 0.05). The D&M method had higher correct diagnosis rate than MSCT or DCEUS alone, the correct diagnosis rate of the D&M method in T1, T2, T3, and T4 staging was significantly higher than that of MSCT (*P* < 0.05). The correct diagnosis rate of the D&M method in T1, T3, and T4 was significantly higher than that of DCEUS (*P* < 0.05).

### 3.5. Results of Validity, Reliability, and Revenue

The results of validity, reliability, and revenue are shown in Table 5. The Youden index of preoperative T1, T2, T3, and T4 staging was 26.76%, 52.61%, 5.74%, and 45.65%, respectively, by the MSCT method; the same was 89.37%, 88.30%, 65.58%, and 76.80%, respectively, by the DCEUS method; and the same was 99.49%, 94.44%, 84.13%, and 90.54%, respectively, by the D&M method, which shows that the D&M method has better validity in preoperative cT staging of GC. The Kappa value of preoperative T1, T2, T3, and T4 staging was 0.382, 0.598, 0.024, and 0.383, respectively, by the MSCT method; the same was 0.823, 0.913, 0.438, and 0.711, respectively, by the DCEUS method; and the same was 0.954, 0.966, 0.707, and 0.881, respectively, by the D&M method. This means the D&M method has better reliability in preoperative cT staging of GC. In addition, the positive predictive value and negative predictive value of the D&M method is highest among the three diagnostic methods, that is to say, the revenue of the D&M method is the best.

## 4. Discussion

The incidence rate of GC is increasing year by year, and surgical treatment is still the first choice. Preoperative accurate staging is very important for the formulation of treatment plan [9]. GC is one of the tumors lacking blood supply; more than 90% of them are adenocarcinoma, and most of them are local thickening and abnormal enhancement of the gastric wall [10]. For tumor tissue, tumor microvessels grow first, and then tumor cells grow and infiltrate [11]. The microvessel perfusion of tumor and peritumoral tissue is consistent, which is different from the surrounding normal tissue structure [12]. Therefore, both MSCT and DCEUS can evaluate the blood supply in tumor. At present, there are some guidelines for preoperative T staging of GC in various staging standards, and the description of imaging features can be used as the basis for the preliminary diagnosis of T staging, but these imaging examination standards are not perfect and need to be further studied.

DCEUS refers to the combination of oral contrast agent and intravenous contrast agent for ultrasound examination [8]. Compared with conventional ultrasound and oral contrast agent ultrasound, it has better contrast and better image quality. In addition, it can dynamically observe and record the perfusion imaging process of the contrast medium in the lesions and normal tissues, so as to improve the diagnostic ability of lesions, so it has great application value in the preoperative cT staging evaluation of GC [13]. However, the study of DCEUS in preoperative evaluation of GC has some shortcomings [14]. Most scholars evaluate lymph node metastasis according to the presence of lymph node metastasis, rather than the number of lymph node metastasis. Therefore, this method needs further study [15].

MSCT is widely used in preoperative evaluation of GC [16]. Compared with conventional CT, it has the following advantages, such as fast scanning, in the abdominal examination, it can reduce the image of respiration and gastrointestinal movement [17]. The image resolution is high. The image can be reconstructed in many directions with high spatial resolution [18]. Through intravenous injection of contrast agent, we can observe the enhancement mode and degree of different tissues and better distinguish the focus tissue and normal tissue [19]. Studies have shown that dynamic contrast-enhanced MSCT in preoperative cT staging of GC has high accuracy and clinical value, but there are also shortcomings, mainly the accuracy of judging the depth of tumor invasion is low [20].

In this study, the correct diagnosis rate of the cT staging of MSCT and DCEUS were compared, in order to find their respective advantages and disadvantages and further study the accuracy of their combined application in preoperative staging of GC, and then some research results were obtained. We found that the correct diagnosis rate of MSCT and DCEUS were 55.34% and 83.98%, respectively, and the D&M method was 93.69%, which was higher than that of MSCT and DCEUS used alone. In addition, the Kappa values of the D&M method in T1 to T4 were 0.954, 0.966, 0.707, and 0.881, respectively, indicating that the consistency of the D&M method in the diagnosis of preoperative T staging of GC is very reliable.

Therefore, with the development of neoadjuvant therapy for GC, it is urgent to find an examination method with high accuracy, less damage, and easy acceptance by patients to evaluate the effect of neoadjuvant therapy. The results of DCEUS and enhanced MSCT in the preoperative cT staging evaluation of GC will lay a foundation for its research in the evaluation of neoadjuvant therapy.

## 5. Conclusion

The dynamic contrast-enhanced MSCT can penetrate the vessel wall. DCEUS is pure blood pool imaging, which can accurately reflect the blood supply of the lesions and dynamically observe the tumor invasion. The application of dynamic contrast-enhanced MSCT improves the accuracy of GC staging and the detection rate of lesions. DCEUS can more accurately predict the cT staging of GC, which has the advantages of nonradiation, simple, repeatable, and noninvasive. The correct diagnosis rate of the DCEUS method for preoperative T stage of gastric cancer was significantly higher than that of the MSCT method. Dynamic contrast-enhanced MSCT combined with DCEUS in the diagnosis of preoperative T staging of GC has more validity, reliability, and revenue than using MSCT or DCEUS alone, which is an image evaluation method worthy of clinical promotion.

## Figures and Tables

**Table 1:** Pathological T staging criteria.

T staging	Infiltration depth
T0	There was no evidence of primary tumor
T1	The tumor invaded the mucosa or submucosa
T2	The tumor infiltrated into the muscularis propria
T3	The tumor penetrated the tissue under serosa, but did not invade the visceral membrane and adjacent structures
T4	The tumor invaded visceral peritoneum or adjacent structures

**Table 2:** Comparison of pathological T staging and MSCT cT staging.

		cT staging results of MSCT					Summation	Correct diagnosis rate (%)
		T0	T1	T2	T3	T4		
pT staging	T1	5	3	3	0	0	11	27.27
	T2	4	1	20	11	0	36	55.56
	T3	0	0	2	8	9	19	42.11
	T4	0	0	0	57	83	140	59.29
Summation		9	4	25	76	92	206	55.34

**Table 3:** Comparison of pathological T staging and DCEUS cT staging.

		cT staging results of DCEUS				Summation	Correct diagnosis rate (%)
		T1	T2	T3	T4		
pT staging	T1	10	1	0	0	11	90.91
	T2	3	32	1	0	36	88.89
	T3	0	0	15	4	19	78.95
	T4	0	0	24	116	140	82.86
Summation		13	33	40	120	206	83.98

**Table 4:** Comparison of pathological T staging and D&M method cT staging.

		cT staging results of combined diagnosis				Summation	Correct diagnosis rate (%)
		T1	T2	T3	T4		
pT staging	T1	11	0	0	0	11	100.00
	T2	1	34	1	0	36	94.44
	T3	0	0	17	2	19	89.47
	T4	0	0	9	131	140	93.57
Summation		12	34	27	133	206	93.69

**Table 5:** Results of validity, reliability, and revenue.

	Validity			Reliability		Revenue	
	Sensitivity (%)	Specificity (%)	Youden index (%)	Coincidence rate (%)	Kappa value	Positive predictive value (%)	Negative predictive value (%)
*MSCT*							
T1	27.27	99.49	26.76	95.63	0.382	75.00	96.04
T2	55.56	97.06	52.61	89.81	0.598	80.00	91.16
T3	42.11	63.64	5.74	61.65	0.024	10.53	91.54
T4	59.29	86.36	45.65	67.96	0.383	90.22	50.00

*DCEUS*							
T1	90.91	98.46	89.37	98.06	0.823	76.92	99.48
T2	88.89	99.41	88.30	97.57	0.913	96.97	97.69
T3	78.95	86.63	65.58	85.92	0.438	37.50	97.59
T4	82.86	93.94	76.80	86.41	0.711	96.67	72.09

*D&M method*							
T1	100.00	99.49	99.49	99.51	0.954	91.67	100.00
T2	94.44	100.00	94.44	99.03	0.966	100.00	98.84
T3	89.47	94.65	84.13	94.17	0.707	62.96	98.88
T4	93.57	96.97	90.54	94.66	0.881	98.50	87.67

## Data Availability

The datasets used and/or analyzed during the current study are available from the corresponding author on reasonable request.
